# Supplementary material for: Characterization of Fusarium Diversity and Head Microbiota Associated with Rice Spikelet Rot Disease
Source: Plants (Basel). 2025 May 20;14(10):1531. doi: 10.3390/plants14101531 (PMC12114772; doi:10.3390/plants14101531)
Supplement: Supplementary file 1 [file plants-14-01531-s001.zip › plants-3571784-supplementary.pdf]

Supplementary Table S1. Results of composite species comparison

Strain	Species complex	Autoploidy (%)
LA01	FIESC	100
LA02	FSAMSC	99.75
LA03	FIESC	99.76
LA05	FIESC	99.76
LA06	FSAMSC	99.51
LA07	FIESC	99.76
LA08	FIESC	99.29
LA09	FIESC	99.29
LA10	FIESC	99.76
LA12	FSAMSC	100
LA14	FSAMSC	93.07
LA17	FIESC	99.06
LA18	FSAMSC	98.78
LA20	FIESC	99.06
LA21	FSAMSC	99.51
LA23	FIESC	99.53
FM01	FSAMSC	100
FM03	FIESC	100
FM04	FIESC	100
FM06	FIESC	100
FM07	FSAMSC	100
FM09	FIESC	99.84

**Strain**	**Species complex**	**Autoploidy** (%)
FM10	FSAMSC	99.64
FM11	FIESC	100
FM13	FSAMSC	99.28
FM14	FIESC	100
FM16	FIESC	100
FM17	FIESC	100
FM19	FSAMSC	99.85
FM20	FSAMSC	99.85
FM21	FIESC	100
FM22	FSAMSC	100
FM23	FIESC	100
FM24	FIESC	100
FR01	FIESC	99.84
FR02	FIESC	100
FR03	FIESC	99.69
FR04	FSAMSC	99.64
FR05	FSAMSC	99.64
FR06	FSAMSC	100
FR07	FSAMSC	100
YR02	FNSC	99.85
YR04	FSAMSC	99.64
YR05	FIESC	100
YR08	FIESC	100
YR09	FIESC	99.53

Strain	Species complex	Autoploidy (%)
YR10	FIESC	100
YR11	FIESC	100
YR12	FIESC	100
YR13	FSAMSC	99.64
YR14	FSAMSC	99.64
YR15	FNSC	100

Supplementary Table S2. Information on reference strains used in this study

Species	Phylogenetic species	Strain number	Host	GenBank no.					
				H3	*CAM*	*TEF1*	*RPB1*	*RPB2*	*TUB2*
F.annulatum	FFSC1	LC13674	Oryza sp	-	MW566362	MW580535	MW024523	MW474481	MW533814
F.annulatum	FFSC2	LC11672	Oryza sp	-	MW566345	MW580518	MW024506	MW474464	MW533797
F.verticillioides	FFSC5	LC5896	Submerged wood	-	MW566336	MW580509	MW024497	MW474455	MW533788
F.verticillioides	FFSC6	LC13653	Glycine max	-	MW566331	MW580504	MW024492	MW474450	MW533783
F.pseudocircinatum	FFSC7	LC13677	Syzygium samarangense	-	MW566370	MW580543	MW024531	MW474489	MW533822
F.pseudocircinatum	FFSC8	LC13676	Syzygium samarangense	-	MW566369	MW580542	MW024530	MW474488	MW533821
F.proliferatum	FFSC9	F026	Musa sp	-	MZ399203	MZ399213	MZ399206	MZ399210	MZ399216
F.fujikuroi	FFSC10	LC13637	Musa nana	-	MW566303	MW580476	MW024464	MW474422	MW533755
F.fujikuroi	FFSC11	LC7147	bamboo	-	MW566319	MW580492	MW024480	MW474438	MW533771
F.asiaticum	FSAMSC1	LC18254	Wheat	OQ124269	-	OQ124899	OQ124550	OQ124842	-
F.asiaticum	FSAMSC2	LC18739	Rice	OQ124435	-	OQ124929	OQ124605	OQ124679	-
F.asiaticum	FSAMSC3	LC18262	Wheat	OQ124276	-	OQ124881	OQ124633	OQ124848	-
F.meridionale	FSAMSC5	LC18704	Maize	OQ124420	-	OQ125043	OQ124524	OQ124706	-
F.graminearum	FSAMSC6	LC18770	Maize	OQ124449	-	OQ125027	OQ124491	OQ124812	-
F.graminearum	FSAMSC7	LC18310	Wheat	OQ124315	-	OQ125019	OQ124579	OQ124788	-
F.boothii	FSAMSC8	LC18779	Maize	OQ124457	-	OQ124948	OQ124518	OQ124776	-
F.vorosii	FSAMSC11	LC15880	Maize	OQ124259	-	OQ124959	OQ124626	OQ124742	-
F.asiaticum	FSAMSC12	LC13789	Oryza sp	-	-	MW620094	MW024661	MW474619	-
F.kyushuense	FSAMSC13	LC18277	Wheat	OQ124288	-	OQ125072	OQ124671	OQ124671	-
F.oxysporum	FOSC1	LC13766	Water	-	-	MW594353	MW024628	MW474586	-

Species	Phylogenetic species	Strain number	Host	GenBank no.					
				H3	*CAM*	*TEF1*	*RPB1*	*RPB2*	*TUB2*
F.curvatum	FOSC2	LC13739	Tulipa gesneriana	-	-	MW594325	MW024600	MW474558	-
F.grosmichelii	FOSC3	JXF4-32	Oryza sp.	-	-	OL771393	OL771377	OL771385	-
F.grosmichelii	FOSC4	M0676	Chamaerops humilis	-	-	OL771396	OL771380	OL771388	-
F.nirenbergiae	FOSC5	LC13752	Hydrangea macrophylla	-	-	MW594338	MW024613	MW474571	-
F.nirenbergiae	FOSC6	LC13754	Olea europaea	-	-	MW594340	MW024615	MW474573	-
F.miscanthi	FNSC1	LC7503	Water	-	-	MW594318	MW024593	MW474551	-
F.commune	FNSC2	LC13823	Musa nana	-	-	MW620161	MW024728	MW474686	-
F.commune	FNSC3	LC11660	Oryza sp	-	-	MW620160	MW024727	MW474685	-
F.paranisikadoi	FNSC4	LC2819	unidentified grass	-	-	MW594315	MW024590	MW474548	-
F.paranisikadoi	FNSC5	LC2823	Pennisetum alopecuroides	-	-	MW594316	MW024591	MW474549	-
F.sulawesiense	FIESC1	TGGF2022_20A	Cucumis melo	-	-	OR427943	OR557322	OR509695	-
F.sulawesiense	FIESC2	LC7939	Capsicum sp.	-	-	MK289641	MK289820	MK289795	-
F.sulawesiense	FIESC3	NRRL34004	human	-	-	GQ505628	HM347167	GQ505806	-
F.sulawesiense	FIESC5	LC13723	Smilax corbularia	-	-	MW594393	MW024578.1	MW474536	-
F.incarnatum	FIESC7	LC13705	Soil	-	-	MW594375	MW024560	MW474518	-
F.luffae	FIESC8	NRRL31167	Human sputum	-	-	GQ505608	-	GQ505786	-
F.luffae	FIESC9	LC13714	Oryza sp	-	-	MW594384	MW024569	MW474527	-
F.luffae	FIESC10	CQ1038	Humulus scandens leaf	-	-	MK289569	MK289870	MK289723	-
F.pernambucanum	FIESC12	LC13715	Heteropogon sp.	-	-	MW594385	MW024570	MW474528	-

Species	Phylogenetic species	Strain number	Host	GenBank no.					
				H3	*CAM*	*TEF1*	*RPB1*	*RPB2*	*TUB2*
F.pernambucanum	FIESC13	LC13716	Gerbera jamesonii	-	-	MW594386	MW024571	MW474529	-
F.pernambucanum	FIESC14	LC13719	Panicum sp.	-	-	MW594389	MW024574	MW474532	-
F.arcuatisporum	FIESC15	LC11639	Oryza sp	-	-	MK289586	MK289798	MK289736	-
F.arcuatisporum	FIESC16	LC13692	Poa annua	-	-	MW594362	MW024547	MW474505	-
F.citri	FIESC17	LC13695	Castanopsis boisii	-	-	MW594365	MW594365	MW474508	-
F.citri	FIESC18	LC4879	Prunus triloba	-	-	MK289615	MK289827	MK289768	-
F.compactum	FIESC19	LC13699	Soil	-	-	MW594369	MW024554	MW474512	-
F.compactum	FIESC20	LC13700	Poa annua	-	-	LC13700	MW024555	MW474513	-
F.guilinense	FIESC21	LC12160	Musa acuminata	-	-	MK289594	MK289831	MK289747	-
F.hainanense	FIESC22	LC11638	Oryza sp	-	-	MK289581	MK289833	MK289735	-
F.humuli	FIESC23	LC13701	Musa nana	-	-	MW594371	MW024556	MW474514	-
F.ipomoeae	FIESC25	CQ1099	Rhododendron x pulchrum	-	-	MK289573	MK289861	MK289727	-
F.ipomoeae	FIESC26	LC6926	Oryza sativa	-	-	MK289619	MK289851	MK289773	-
F.irregulare	FIESC27	LC12146	Bamboo	-	-	MK289583	MK289865	MK289738	-
F.irregulare	FIESC28	LC13712	Digitaria sp	-	-	MW594382	MW024567	MW474525	-
F.lacertarum	FIESC29	LC7927	Capsicum sp	-	-	MK289637	MK289866	MK289791	-
F.lacertarum	FIESC30	LC7931	Capsicum sp	-	-	MK289638	MK289867	MK289792	-
F.nanum	FIESC31	LC12168	Musa acuminata	-	-	MK289602	MK289871	MK289755	-
F.nanum	FIESC32	LC1385	Solanum lycopersicum	-	-	MK289612	MK289873	MK289765	-
F.concolor	FCOSC1	NRRL 13459	-	-	-	GQ505674	MH742523	GQ505852	-
